# Characterization of Biofilm Formation by *Borrelia burgdorferi In Vitro*

**DOI:** 10.1371/journal.pone.0048277

**Published:** 2012-10-24

**Authors:** Eva Sapi, Scott L. Bastian, Cedric M. Mpoy, Shernea Scott, Amy Rattelle, Namrata Pabbati, Akhila Poruri, Divya Burugu, Priyanka A. S. Theophilus, Truc V. Pham, Akshita Datar, Navroop K. Dhaliwal, Alan MacDonald, Michael J. Rossi, Saion K. Sinha, David F. Luecke

**Affiliations:** 1 Lyme Disease Research Group, Department of Biology and Environmental Sciences, University of New Haven, West Haven, Connecticut, United States of America; 2 Department of Physics, University of New Haven, West Haven, Connecticut, United States of America; Université d’Auvergne Clermont 1, France

## Abstract

*Borrelia burgdorferi*, the causative agent of Lyme disease, has long been known to be capable of forming aggregates and colonies. It was recently demonstrated that *Borrelia burgdorferi* aggregate formation dramatically changes the *in vitro* response to hostile environments by this pathogen. In this study, we investigated the hypothesis that these aggregates are indeed biofilms, structures whose resistance to unfavorable conditions are well documented. We studied *Borrelia burgdorferi* for several known hallmark features of biofilm, including structural rearrangements in the aggregates, variations in development on various substrate matrices and secretion of a protective extracellular polymeric substance (EPS) matrix using several modes of microscopic, cell and molecular biology techniques. The atomic force microscopic results provided evidence that multilevel rearrangements take place at different stages of aggregate development, producing a complex, continuously rearranging structure. Our results also demonstrated that *Borrelia burgdorferi* is capable of developing aggregates on different abiotic and biotic substrates, and is also capable of forming floating aggregates. Analyzing the extracellular substance of the aggregates for potential exopolysaccharides revealed the existence of both sulfated and non-sulfated/carboxylated substrates, predominately composed of an alginate with calcium and extracellular DNA present. In summary, we have found substantial evidence that *Borrelia burgdorferi* is capable of forming biofilm *in vitro.* Biofilm formation by *Borrelia* species might play an important role in their survival in diverse environmental conditions by providing refuge to individual cells.

## Introduction

*Borrelia burgdorferi,* the causative agent of Lyme disease, is known to employ a variety of mechanisms to counteract eradication by its host, including the adoption of several alternate morphologies in response to changing environmental conditions [1]–[5]. In addition to its familiar corkscrew-shaped spirochete form, *Borrelia burgdorferi* can transform from motile spirochetes into cystic, granular, or cell wall deficient forms in the presence of various unfavorable environmental conditions [6]–[9]. For example, cystic forms can be induced by unfavorable conditions such as nutrition deprivation, high ambient pH, or adverse temperature [2] [6] [5]. It has also been demonstrated that cystic forms are able to revert to vegetative spirochetes *in vitro* [1] and *in vivo* [3], suggesting that these alternative formations are one of the ways that *Borrelia burgdorferi* survives in otherwise unfavorable conditions. Besides the cystic form, agglomeration of spirochetes into organized aggregates, containing numerous cystic forms, granules as well as spirochetes, has been also observed both *in vitro* and *in vivo* [5] [10]–[16].

The hypothesis investigated in this study is that *Borrelia burgdorferi* aggregates are actually a purposeful, functional biofilm. Biofilms are complex aggregations of planktonic microorganisms that serve to protect the resident individuals from hostile environments [17]–[18]. Bacterial biofilms can be induced by extreme, nonphysiologic pH, or extreme temperature or by addition of high concentration of metals as well as addition of xenobiotics, antimicrobial agents and even oxygen in some species [19]–[20].

To be able to develop high resistance to environmental stressors or therapeutic interventions, biofilms create unique, complex structures, which are covered with a protective layer consisting of a mixture of extracellular polymeric substances (EPS) secreted by the cells established within the biofilm [20].

If *Borrelia burgdorferi* is capable of forming a true biofilm, this may explain why these bacteria were capable of withstanding different adverse environmental conditions as reported in several *in vivo* and *in vitro* studies [2] [5]–[6] [21]–[28]. Despite the potential importance of this hypothesis, to date there have been no studies that have attempted to determine whether *Borrelia* is indeed capable of biofilm formation. We have previously suggested that biofilm might provide another powerful survival mechanism for *Borrelia* spirochetes based on observations of microscopic images of *Borrelia* aggregates [29].

In this study, we employ additional approaches to investigate whether *Borrelia burgdorferi* can form biofilm *in vitro*. *Borrelia burgdorferi* aggregates were evaluated for several biofilm-specific characteristics, such as morphological heterogeneity and rearrangement, secretion of an EPS matrix, presence of pronounced levels of extracellular DNA and calcium within the aggregates, as well as preference for various surfaces and substrate matrices.

## Results

### Description of the Different Stages of *Borrelia burgdorferi* Aggregate Formation with Various Modes of Microscopy

**Figure 1:**
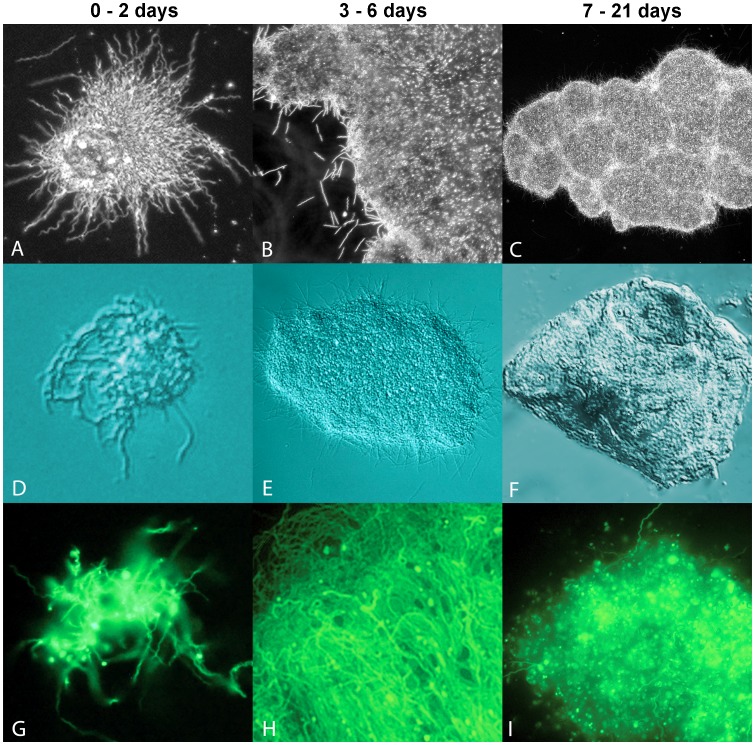
Representative images of *Borrelia burgdorferi* B31 strain aggregates in the early (1^st^ column, 0 to 2 day), middle (2^nd^ column, 3 to 6 days) and late (3^rd^ column, 7 to 21 days) stages of development, observed with dark field (A, B, C – 400× magnification); differential interference contrast (D, E, F - 400× magnification); and FITC-band epifluorescence (G, H, I – 400× magnification). D, F, and I are extended depth of field composites.

The aggregate formation of *Borrelia burgdorferi* was observed and analyzed at different cell densities using optical and atomic force microscopy (AFM) methods. As previously reported for *Borrelia burgdorferi* [15], in early log phase cultures (1−5×10^6^ cells/ml) almost all cells are individual spirochetes, but as the concentration increases above 1×10^7^ cells/ml, cell aggregates start to form. The different stages of aggregate formation of representative cultures of B31 and B31-GTP strains of *Borrelia burgdorferi,* cultured in polystyrene tubes with no additional matrix provided, were examined. Figure 1 demonstrates that with an initial cell concentration of 1×10^7^ cells/ml, aggregates were observed in these cultures under all modes of microscopy used in early (column 1: days 0–2), middle (column 2, days 3–6) and late stages (column 3, days 7–21). With dark field illumination, spirochetes were observed arrayed around the periphery of a semi-spherical core that diffused light strongly and heterogeneously within a day (Figure 1A). In middle-stage samples representing 3–6 days of culturing, the matrix adopted a more filmic aspect and appeared to be pliant, with a relatively low viscosity allowing the film to flatten under the weight of the coverslip (Figure 1B). Moderate densities of spherical elements were visible within the matrix, but dark field illumination did not permit observation of matrix-embedded spirochetes. In late stage structures, representing day 7–21 days (Figure 1C), the matrix exhibited higher rigidity, with hills, valleys and cracks observed in the structures. Using Differential Interference Contrast (DIC) microscopy, the heterogeneity of the film’s composition was more readily visible. The grainy appearance of the film under DIC (Figure 1E, F) indicated the film contained constituents of non-uniform optical density. The late stage sample (Figure 1F) exhibited substantial rigidity, resisting even applied compression of the cover slip. With FITC-band illumination of the GFP-expressing hybrid strain, spirochetes involved in initial formation of the matrix were clearly visible (Figure 1G). Middle-stage colonies were largely composed of now-visible spirochetes (Figure 1H), whereas in late-stage colonies, round bodies predominated (Figure 1I). Figure S1 is a dark field microscopy image showing aggregates developed in a roughly circular manner, with circles apparently converging to form larger biofilm-like structures. Video S1 is a movie file showing two large floating *Borrelia burgdorferi* aggregates actively growing surrounded by individual spirochetes. *Borrelia burgdorferi* strain 297 showed similar growth at all culture times (data not shown).

### Matrix Requirement of *Borrelia burgdorferi* Aggregates

While aggregate formation did not require any special surface for *Borrelia burgdorferi*, especially when the concentration exceeding 1×10^7^ cells/ml, we investigated the possibility that this pathogen may be capable of forming aggregate structures on solid surfaces at lower cell concentrations. The first step of sessile biofilm formation involves planktonic bacterial cells or cell aggregates attaching to either abiotic (glass, plastic) or biotic surfaces (plant or animal matrices). Therefore, any difference in the aggregate attachment and/or growth on various surfaces was examined. The various surfaces included abiotic surfaces (glass and polystyrene laboratory plastic), and biotic surfaces (Matrigel, rat tail collagen type I, fibronectin, hyaluronan, laminin, and agarose). B31 and 297 strains of *Borrelia burgdorferi* cells were plated on either uncoated or various matrix-coated 48-well tissue culture plates and incubated for up to 7 days at 33°C with 5% CO_2_. The resulting colonies were stained and quantified with crystal violet. *Borrelia burgdorferi* B31 strain attached and grew on every surface tested with as little as 5×10^3^ cells/ml. Visible colonies can be seen as early as 2 days of culture on all surfaces studied with greater than 1×10^6^ cells /ml. The aggregate formation on all surfaces also showed concentration dependence after 7 days of culture (Figure S2). *Borrelia burgdorferi* strain 297 showed similar growth on all matrices tested (data not shown). Figure S3 shows representative images of *Borrelia burgdorferi* strain B31 aggregate formation on biotic and abiotic surfaces.

### Internal Morphological Rearrangement during *Borrelia burgdorferi* Aggregate Development

**Figure 2:**
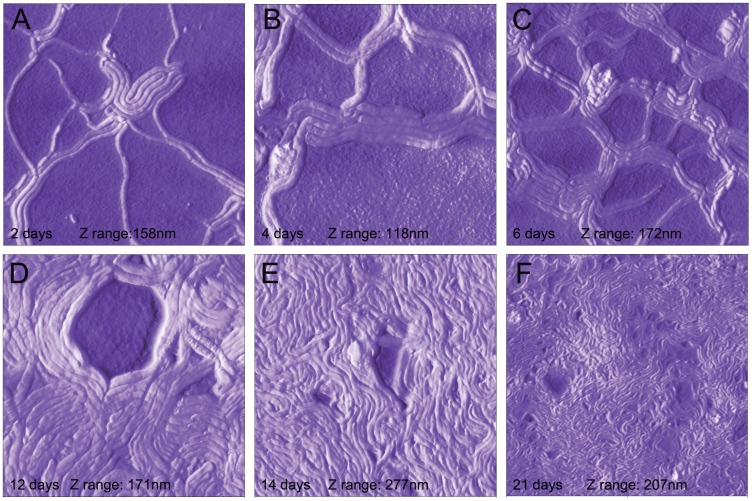
Three-dimensional AFM images of aggregate development of *Borrelia burgdorferi* B31 strain on agarose substrate after 2 days (A), 4 days (B), 6 days (C) 12 days (D) 14 days (E) and 21 days (F). The preparation of *Borrelia burgdorferi* cells on agarose-coated mica discs is described in Material and Methods. All scans were scanned at 0.4 Hz using contact mode and the individual Z (height) ranges are indicated on the panels. The images were produced and measurements determined with NanoRule© software.

**Figure 3:**
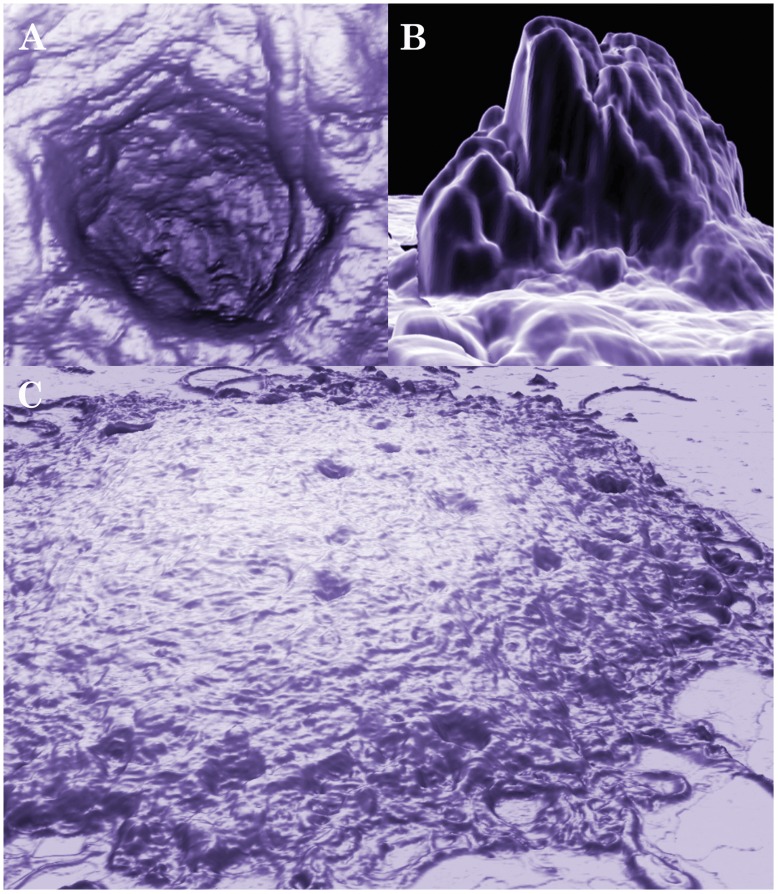
Three-dimensional AFM images of a mature aggregate of *Borrelia burgdorferi* B31 strain after 20 days. The preparation of *Borrelia burgdorferi* cells on mica is described in the Materials and Methods. The scan was conducted with 0.4 Hz using contact mode. A and B show a pit and a protrusion, respectively, of a large mature aggregate as depicted in C. Images A and C were produced with NanoRule© software; image B was produced with a custom meshing utility and MeshLab open-source software.

**Figure 4:**
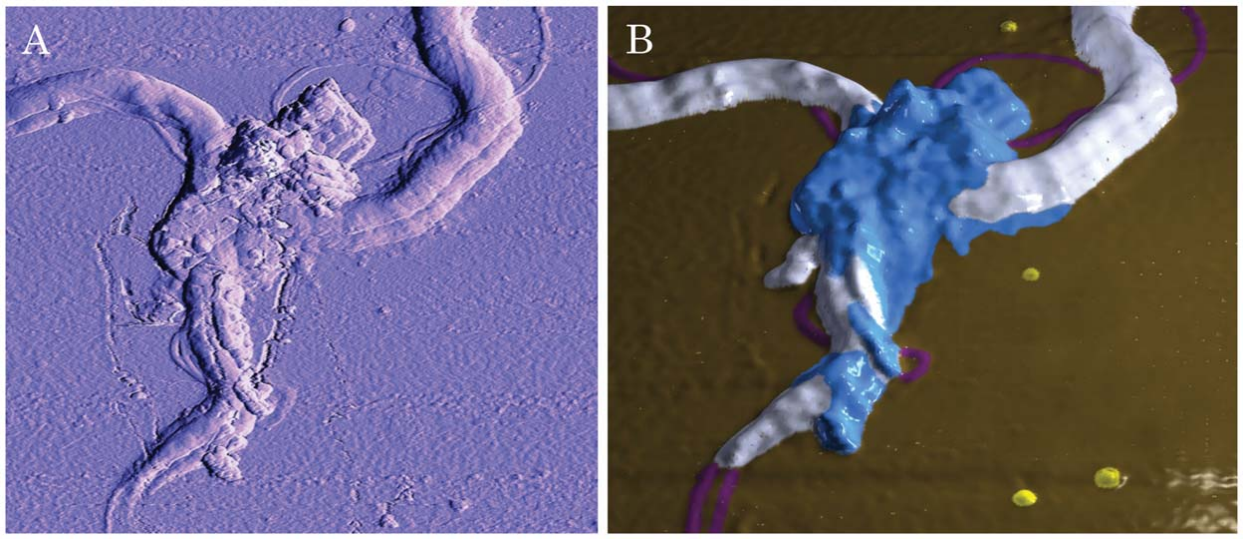
Three-dimensional AFM images of *Borrelia burgdorferi* B31 strain early aggregates on agarose substrate. The preparation of *Borrelia burgdorferi* cells on mica is described in the Materials and Methods. The sample was scanned at 0.3 Hz using contact mode. A: The original AFM image produced with NanoRule© software. B: The AFM dataset was converted to a 3D mesh via a custom meshing utility, cleaned with MeshLab open-source software, then imported to Adobe Photoshop and false-color hand-painted with 3D painting tools. White: spirochete bodies; blue: potential EPS matrix; purple: protrusions; and yellow: small round bodies.

Internal morphological rearrangement during aggregate development is one of the hallmark features of biofilm [20]. In order to investigate the possibility that these aggregates have internal morphological rearrangement, we utilized the relatively new microscopic technology, Atomic Force Microscopy (AFM). Unlike electron microscopy, AFM does not require sample fixation, thus allowing monitoring of morphological rearrangements in live *Borrelia burgdorferi* aggregates on different surfaces at different time points at an unprecedented nanometer scale. To be able to visualize live *Borrelia burgdorferi* colonies, we used a recently developed AFM hydrogel method [30] wherein mica surfaces are coated with different matrices (collagen, fibronectin and agarose), treated with the bacterial culture, and observed via AFM at different time points. Out of the three substrates tested, agarose provided the most useful images, probably due to the relatively smooth agarose surface [30]. *Borrelia burgdorferi* cells were found partially embedded in agarose, with the upper half of the cells exposed to the air and the AFM probe (Figure 2). Inspection of the cells revealed that the outer membrane of most spirochetes appeared intact. *Borrelia burgdorferi* on agarose surfaces retained their spirochete form, but readily associated with other spirochetes as early as within two days, and most spirochetes were found to pair (Figure 2A–C). The pairs became progressively more organized, forming a highly structured mesh of aggregates resembling a web. Some spirochetes folded on themselves, usually found in the center of aggregations (Figure 2D). Cystic forms were also incorporated into the aggregate superstructure, and were found evenly throughout the aggregates. Aggregates and meshes were incorporated into a larger network over time (Figure 2E and F). The network contained many curved arches and loops that were of similar size, suggesting high levels of organization. Structural rearrangements continued as the aggregates matured further, eventually resulting in deep pits and tall protrusions with currently unknown functions (Figure 3 and Video S2). An AFM scan made in contact mode of a pair of *Borrelia burgdorferi* spirochetes initiating aggregation was acquired (Figure 4A). As aspects of this scan were difficult to interpret from the conventionally processed image, the raw AFM data set was also converted to XYZ file format, meshed and rectified via MeshLab open-source software, then imported to Adobe Photoshop and false-color hand-painted with 3D painting tools (Figure 4B, white: spirochete bodies; blue: potential matrix; purple: several filament like protrusions; and yellow: small round bodies). The 3D mesh image revealed that at the junction, there is a raised area of pebble-textured material that distinctly differs from relatively smooth-textured spirochete bodies further from the junction. There are also small round bodies located in the proximity of the aggregates. A protrusion forming a loop can also be seen in the acute angle of the junction, suggesting the possibility that such loops may play a role in shaping or reinforcing junction structure.

These protrusions are commonly seen in other AFM images in early spirochete aggregates; in some cases, significant numbers of these protrusions can be seen (Figure S4).

### Extracellular Polysaccharides on the Surface of the *Borrelia burgdorferi* Aggregates

**Figure 5:**
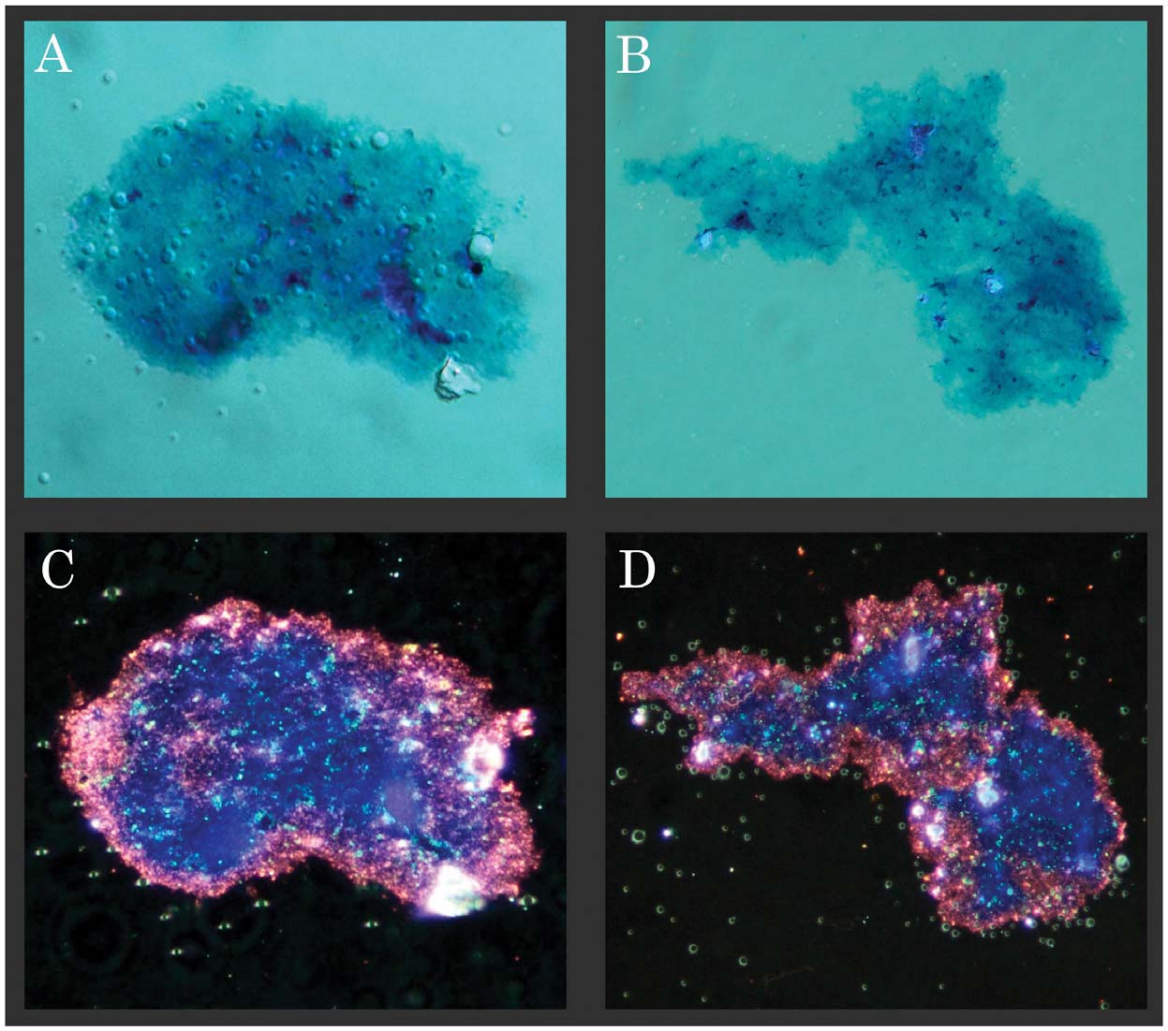
Representative image showing Spicer & Meyer aldehyde fuchsine-Alcian blue sequential staining pattern of two *Borrelia burgdorferi* B31 strain aggregates by differential interference contrast (A and B) and dark field microscopy (C and D). Fuchsia coloration is indicative of weakly acidic sulfomucin; purple coloration indicates strongly acidic sulfomucins and/or sulfated proteoglycans; blue coloration indicates non-sulfated, carboxylated mucins. 500× magnification.

Study of the chemical properties of the substance covering the colonies was needed to determine presence of true biofilm. Demonstration of extracellular polysaccharides covering *Borrelia burgdorferi* aggregates similar to other known biofilms would provide strong evidence for the existence of *Borrelia* biofilms [20]. For these experiments, an adaptation of the Spicer & Meyer aldehyde fuchsine-Alcian blue sequential staining method was used. The Spicer & Meyer sequence differentiates between sulfated and non-sulfated/carboxylated mucins. Fuchsia coloration is indicative of weakly acidic sulfomucins; purple coloration indicates strongly acidic sulfomucins and/or sulfated proteoglycans; blue coloration indicates non-sulfated/carboxlated mucins. *Borrelia burgdorferi* strain B31 aggregates imaged with DIC (Figure 5A and B) and dark field microscopy (Figure 5C and D), showed strong staining for both sulfated and non-sulfated/carboxylated mucins. The micrographs revealed that the periphery of the films stained fuchsia, indicating sulfomucin content (some portions stained deep purple, possibly indicating proteoglycan composition). Near the center, areas of the films stained predominately blue, indicating non-sulfated/carboxylated polysaccharide mucin composition similar to bacterial alginate. The green-tinged spherical bubbles are most likely water inclusions in the Permount mounting agent resulting from incomplete film dehydration prior to mounting.

**Figure 6:**
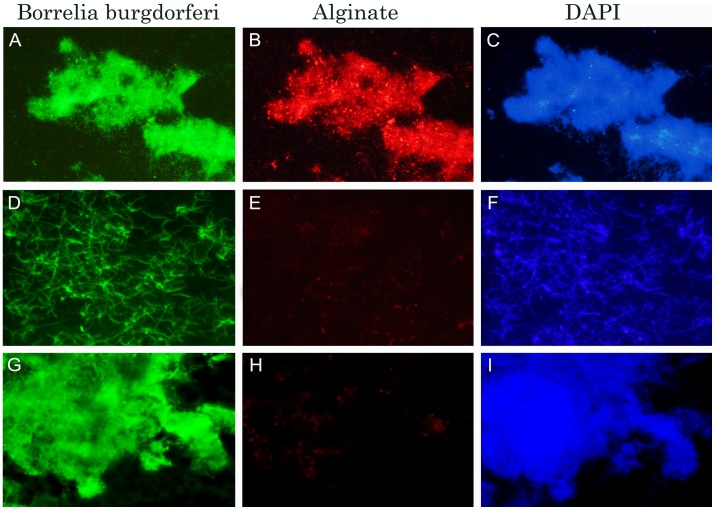
Immunohistochemical staining of a collagen-embedded *Borrelia burgdorferi* B31 aggregates (Panels A–C and G–I) and individual spirochetal cells (Panels D–F) for alginate (red staining; Panels B andI) and *Borrelia* antigen (green staining; Panels A,D,G) expression using fluorescent microscopy (see methods for detailed protocol). Panel H shows the lack of red staining in the absence of the primary antibody for alginate. Panels C, F and I show DAPI - DNA counterstain images. 400× magnification.

Because these results highly suggested that the secreted substance covering the aggregates was largely composed of alginate or a chemically similar compound, the experiments were repeated using immunohistochemical methods with anti-alginate and anti-*Borrelia* antibodies (Figure 6A–E). The immunohistochemical data showed that *Borrelia burgdorferi* B31 aggregates stained strongly with both anti-alginate (red staining, Figure 6B) and anti-*Borrelia* antibody (green staining, Figure 6A and G). Planktonic spirochetes only stained with anti-*Borrelia* antibody but not with alginate (Figure 6D, E). To depict the size and the form of aggregates and planktonic spirochetes, DAPI counterstained images (Figure 6C, F, I) have also been included. As a negative control, the primary alginate antibody was omitted from the immunohistochemical method and replaced with normal rabbit sera (see Materials and Methods). This experiment indicates that *Borrelia burgdorferi* B31 aggregates did not show any staining in the absence of the primary alginate antibody (Figure 6H). To further demonstrate the staining pattern of alginate on the surface of *Borrelia burgdorferi* aggregates, but not on the individual spirochetes, we have included an additional image in the supplemental information section, showing the surface of the two aggregates, but not surrounding spirochetes, stained strongly with anti-alginate antibody. (Figure S5).

### Calcium Presence on the Surface of the *Borrelia burgdorferi* Aggregates

**Figure 7:**
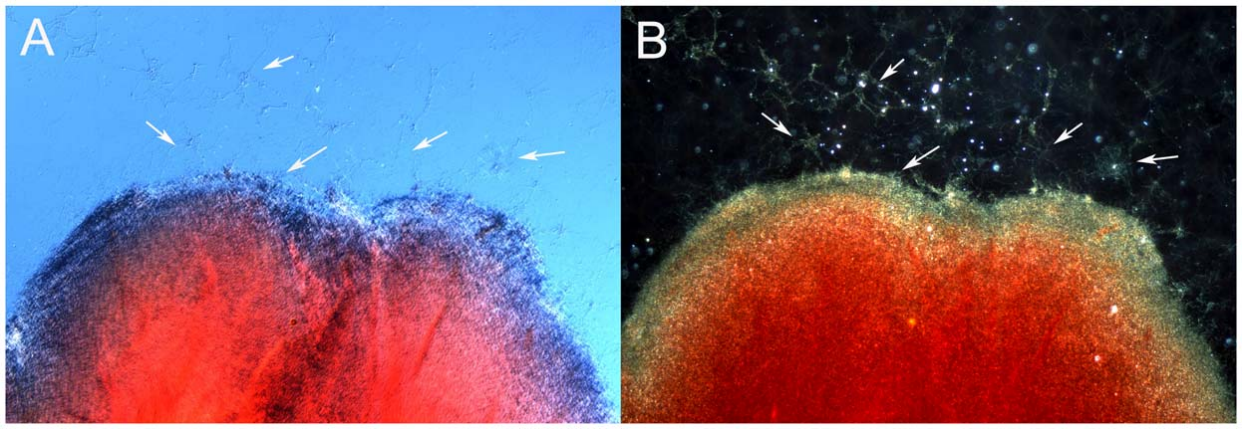
*Borrelia burgdorferi* B31 strain large aggregate surrounded by individual spirochetes and several small aggregates stained with the calcium-detecting stain Alizarin. Red coloration indicates presence of calcium, by differential interference contrast (Panel A) and dark field microscopy (Panel B). White arrows indicate unstained spirochetes and small aggregates. 400× magnification.

Alginate is known to freely associate with calcium to form an insoluble calcium alginate. Therefore a large *Borrelia burgdorferi* aggregate surrounded by individual spirochetes and small aggregates were analyzed for potential calcium content, using a calcium specific staining method with Alizarin Red-S stain. The center of a large *Borrelia burgdorferi* aggregate acquired the strong red coloration indicative of significant calcium presence as depicted by DIC (Figure 7A) and dark field (Figure 7B) microscopy methods, but the periphery of the large aggregate did not, nor did the proximate individuals and smaller aggregates. Unstained dispersed spirochetes, the small aggregates and the periphery of the large aggregate are marked with white arrows (Figure 7A and B).

### Extracellular DNA Presence on the Surface of the *Borrelia* Aggregates

**Figure 8:**
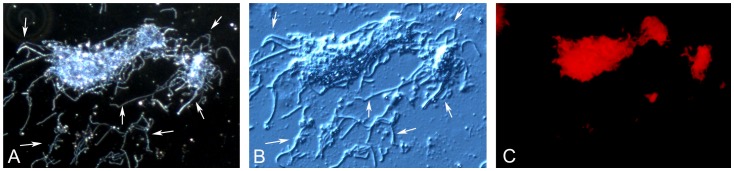
*Borrelia burgdorferi* B31 aggregates surrounded by individual spirochetes (marked with white arrows) stained with the DDAO [7-hydroxy-9H-(1, 3-dichloro-9, 9 dimethylacridin-2-one DNA binding fluorescent dye. A: Dark field image B: Differential interference contrast image, and C: DDAO red stained fluorescent image of the same cellular structures. 400× magnification.

Finally, *Borrelia burgdorferi* aggregate surfaces were examined for the presence of another biofilm marker, extracellular DNA (eDNA), using nucleic-acid-specific dyes. eDNA is known to have several roles in biofilm development, from substrate attachment to stabilization of the extracellular matrix [31]. *Borrelia burgdorferi* B31 cells were cultured for 7 days from a starting concentration of 1×10^7^cells/ml and were stained with 7-hydroxy-9H-(1, 3-dichloro-9, 9 dimethylacridin-2-one (DDAO) red fluorescent dye, commonly used for eDNA detection [32]–[33]. *Borrelia burgdorferi* aggregates, but not the surrounding individual spirochetes (marked with white arrows) were found to contain abundant, dispersed double-stranded DNA (red staining, Figure 8C). To depict the size and the morphology of the aggregates and spirochetes, dark field and DIC images were included (Figure 8A and B respectively).

## Discussion

In this paper, we provide substantial evidence that features of *Borrelia burgdorferi* aggregate development correspond to well-established characteristic features of biofilm formation. The progression of biofilm formation by *Borrelia burgdorferi* closely resembles that of other biofilms described in literature [20]. This starts by either creating floating biofilms or by adherence of individual cells to biotic or abiotic surfaces, followed by a significant expansion of the aggregates into a three-dimensional structure. Critically, the colonies develop a protective EPS layer. The EPS matrix largely consists of alginate, the primary polymeric compound in the EPS of other well-studied bacterial biofilms [20]. Furthermore, there is calcium and eDNA in the matrices of mature colonies. In addition, we have demonstrated that dynamic rearrangements occur within the aggregate structure, beginning early in development, and that continued rearrangement culminates in a complex, highly heterogeneous morphology.

Among the order Spirochaetales, *Treponema denticola* and *Leptospira spp* have already been reported to able to participate in biofilms [34]–[36]. *Treponema denticola* biofilms, for example, were found in periodontal diseases, where *Treponema denticola* was part of polymicrobial biofilms. Further investigation of *Treponema denticola* biofilm development revealed that it could be achieved *in vitro* also, on fibronectin surfaces in a low-shear-force environment [34]. Our optical microscopy and AFM results demonstrated that *Borrelia burgdorferi* can also attach to different matrix surfaces, including fibronectin, but the attachment would only occur in a static or low-shear-force environment. *Leptospira spp* were identified first in polymicrobial biofilm in dental water systems [36]. Recently, *Leptospira* biofilms were also characterized by an *in vitro* study [35], and were found to preferentially attach to glass and polystyrene surfaces in a static environment, with some of species able to produce floating biofilms. We have demonstrated here that *Borrelia burgdorferi* can also attach to abiotic surfaces and form biofilm colonies, and we have also observed free-floating biofilm colonies without any apparent surface attachment.

To characterize the detailed structural components of the *Borrelia burgdorferi* biofilm in high resolution, we have opted to use AFM instead of electron microscopy. For electron microscopy, the samples must be prepared by fixation or cryopreservation prior to imaging under high vacuum, which may alter the true morphology of the biofilm; also, the development of a single biofilm structure cannot be observed over time. Using AFM, we were able to show the sequential steps of the initial adhesion event and the different morphological changes over a 2-week time period with unprecedented resolution. We have also shown evidence of potential EPS secretion in early aggregates.

Our AFM images of *Borrelia burgdorferi* aggregates demonstrated channel-like structures, which have been reported to be observed in other biofilms [37], including *Leptospira spp* biofilms [36]. The previous biofilm studies suggested that those channels provide oxygen and nutrient diffusion to embedded cells as well as waste removal [37].

Our contact mode AFM results also suggested that there is a substance developing on the aggregate surface at an early stage. Data from the literature [38] confirms that that chemical substances secreted by the cells in biofilm are diverse, including polysaccharides, proteins, nucleic acids, glycoproteins, and phospholipids. We identified some of these components in the extracellular substance of *Borrelia burgdorferi* colonies, such as sulfated and non-sulfated mucopolysaccharides, dispersed eDNA, and even embedded calcium. Using an immunohistochemical technique, we determined that the copious non-sulfated mucopolysaccharide present is largely alginate. Bacterial alginate, an O-acetylated linear polymer of β-D-mannuronate and α-L-guluronate residues, is mainly known as a component of *Pseudomonas aeruginosa* [39] and *Azotobacter vinelandii* [40] biofilms; however, it was recently reported that *Leptospira biflexa* biofilm also contains alginate [35]. These microorganisms are significantly different taxonomically, yet still produce identical or nearly identical types of some extracellular compounds in their biofilms. They even share common metabolic pathways for alginate production, regulated by clustered genes; including *algA*, a gene-encoding GDP-mannose pyrophosphorylase*; algD*, encoding GDP-mannose dehydrogenase; and *algE*, coding for a membrane protein involved in alginate export [41]. Additional genes involved in the alginate expression process also were identified by chromosomal co-location [41]. Our extensive similarity searches for potential homological genes in *Borrelia spp* for alginate production using the BLAST search engine from the NCBI website yielded no significant matches. One potential explanation was provided from a study where a *Pseudomonas aeruginosa* mutant strain possessed known deficiencies for several enzymes of the alginate biosynthesis, yet was still capable of producing alginate [42]. The study showed that the enzymes of the Entner-Doudoroff pathway can also be involved in the synthesis of alginate from glucose or gluconate, and it was proposed that glyceraldehyde-3-phosphate was a precursor in polymer biosynthesis [43]. *Borrelia burgdorferi* appears to possess only some of the enzymes of the Entner-Doudoroff pathway; the cells may be employing a modified version of this pathway, or synthesizing alginate via another yet unknown alternative pathway. Identification of the exact metabolic pathways involved in alginate production for *Borrelia burgdorferi* might help to better understand how this form can develop.

In this study, we have also provided evidence that the surface of the aggregates, but not the surface of the surrounding individual spirochetes, contains significant amounts of eDNA and calcium. eDNA is now widely accepted to be one of the major components of the biofilm matrix of many bacteria and has been shown to perform multifaceted functions in bacterial biofilms, such as maintaining architectural integrity and enhancing resistance to environmental stressors [31]–[33]. A recent study showed that negatively charged extracellular DNA can chelate cations, including magnesium and calcium, and induce the expression of genes involved in the modification of cell surface components and genes involved in antimicrobial resistance in *Pseudomonas aeruginosa* [32]. In our future studies, we will investigate whether the previously observed increase in resistance of *Borrelia* aggregates to environmental stressors is due to similar interactions of the cationic environment and eDNA component of EPS.

The next very important question to be addressed is whether *Borrelia burgdorferi* biofilm exists *in vivo* and potential function of those structures might serve other than shelter individual spirochetes from environmental stressors. Borrelia aggregates were recently demonstrated in the midguts of naturally-infected nymphs during their blood meal and it was suggested that aggregates might have a role in successful transmission of *Borrelia* [16]. It was reported that aggregating spirochetes have undergone complex morphological and developmental changes especially in the initial adherence phase, during which non-motile spirochetes advance as networks toward the basolateral surface of the gut epithelium [16]. We will reexamine those *Borrelia* aggregates in the midguts of feeding nymphal and adult *Ixodes scapularis* ticks with several different immunohistochemical techniques, including biofilm-specific markers, using a high-speed, high-sensitivity camera capable of rapidly acquiring the requisite image sets for three-dimensional visualization.

In summary, our study is the first to characterize the biofilm formation by *Borrelia* species *in vitro*. Biofilm formation by *Borrelia* species might play an important role in their survival in diverse environmental conditions. Further characterization of this form will help us to better understand the different survival strategies of *Borrelia burgdorferi*.

## Materials and Methods

Low passage isolates of B31 and 297 strains of *Borrelia burgdorferi* were obtained from American Type Tissue Collection. A genetically engineered fluorescent B31 strain of *Borrelia burgdorferi* (B31 GFP) was received from Dr. G. Chaconas, (University of Calgary) [44]. All wild type *Borrelia burgdorferi* strains were cultured in BSK-H media, with 6% rabbit serum (complete media from Sigma-Aldrich #B8291), without additional antibiotics that may adversely influence morphological development. The fluorescent B31 strain was cultured with the additional of gentamicin at 100 µg/ml (Sigma-Aldrich) in BSK-H complete media in order to prevent the loss of the GFP tag. All cultures were incubated at 33°C with 5% CO_2_. The stock cultures were maintained in sterile 15 ml glass tubes, without agitation. To establish spirochete control cultures to initiate all aggregate experiments, *Borrelia burgdorferi* cells were cultured in a shaking incubator at 33°C and 250 rpm in glass culture tubes at a concentration less than 5×10^6^ cells/ml. Under those culture conditions, no aggregate formation was observed, and the culture contained a homogenous spirochete population [25]. To initiate aggregate growth, 1×10^7^ cells/ml suspensions of *Borrelia* cultures in BSK-H complete media were either placed in small 2 ml cryo-vial tubes (1.8 ml suspension/tube, Fisher Scientific), or were grown on either uncoated or various matrix-coated 48-well tissue culture plates (1 ml suspension/well, Fisher Scientific), or were grown on uncoated 2-well polystyrene or glass chamber slides (2 ml suspension/well, Thermo-Scientific). The coating of the 48-well tissue culture plates was performed with 0.2 ml of either Matrigel (BD Biosciences, diluted 1∶1 with BSK-H complete media) collagen rat tail I (0.01 mg/ml, Sigma), laminin (0.05 mg/ml, Sigma), hyaluronan (0.05 mg/ml, Sigma) fibronectin (0.05 mg/ml Sigma) or 2% Seakem^TM^ agarose (Fisher Scientific). The plates were placed ajar in a tissue culture hood for 2 hours at room temperature. The excess material was aspirated and the wells were washed twice with phosphate buffered saline (PBS) pH 7.4, left ajar for 1 hour to dry in a sterile laminar flow hood, then covered and stored at 4°C until use.

### Microscopy Techniques

Specimens for optical imaging were examined at different time points on a Leica DM2500 biomedical microscope equipped with dark field, differential interference contrast (DIC) and epifluorescent illumination, and recorded with either a Leica DFC500 microscope camera or a Canon 35 mm DSLR camera with a Martin SLR adapter. Extended depth of field images were assembled with CombineZP open source image processing software.

For atomic force microscopy scanning, *Borrelia burgdorferi* cultures were centrifuged at 8000×g for 10 minutes at room temperature and the resultant cell pellets were resuspended in 0.1 M PBS pH 7.4. The suspension was dried onto a 10 mm mica disc (Ted Pella V1 Cat #50) coated with 2% Seakem^TM^ agarose (Fisher Scientific), or was dried onto an uncoated mica disc, rinsed with double distilled water, and dried again at room temperature. The discs were then scanned on a Pacific Nanotechology AFM using low-spring-constant silicon contact probes for maximum surface relief. Images were processed and measurements obtained using NanoRule© image processing software.

### Crystal Violet, LIVE/DEAD Baclight and Extracellular DNA Staining Methods

*Borrelia burgdorferi* colonies were visualized and the mass quantified by standard crystal violet method as described previously [25]. Care was taken to ensure that biofilm was not removed during the washing steps. Crystal violet stain was released from the cells with 95% ethanol and the absorbance was read at 595 nm for quantitative results.

For visualization of the live and dead cells in the colonies, the LIVE/DEAD BacLight Bacterial Viability Assay kit (Invitrogen) was utilized, following the manufacturer’s instructions. Images were acquired by fluorescent microscopy.

To visualize the extracellular DNA on the surface of the aggregates and individual spirochetal cells, 1×10^7^ cells/ml suspensions of *Borrelia burgdorferi* cells were cultured on collagen-coated 4-well chamber slides (see above for slide preparation) for 7 days. The resulting aggregates were washed twice with TE buffer pH 8.0 and the extracellular DNA of the unfixed aggregates was stained with 1 µM DDAO [7-hydroxy-9H-(1, 3-dichloro-9, 9 dimethylacridin-2-one)] for 30 min at 37°C in the darkened environment. DDAO-treated samples were then washed twice, 3 minutes per wash, in TE buffer. Slides were mounted in PermaFluor aqueous mounting medium (Thermo Scientific) and coverslipped in a darkened environment. DDAO was substituted with TE buffer pH 8.0 as a negative control.

### Spicer & Meyer Staining

The staining method was performed as described previously with minor modifications [45]. Briefly: *Borrelia burgdorferi* aggregates were grown as described above for 7 days and were fixed on microscope slides with pre-chilled 1∶1 acetone/methanol mixture at −80°C for 5 min. The colonies were then stained with aldehyde fuchsine solution (Sigma-Aldrich, 0.5% fuchsine dye, 6% acetaldehyde in 70% ethanol with 1% concentrated hydrochloric acid) for 20 minutes. After dipping the slides in 70% ethanol for 1 minute, the slides were rinsed with double-distilled water for 1 minute, then the colonies were sequentially stained with 1% Alcian blue 8GX (Sigma-Aldrich, dissolved in 3% acetic acid, pH 2.5) for 30 minutes. The slides were rinsed with double distilled water for 3 minutes and dehydrated in chilled graded ethanol washes (50%, 70%, and 95%, 3 minutes each), then dipped in chilled xylene for 2 minutes and mounted with Permount media (Fisher Scientific).

### Immunohistochemistry

Detection of alginate expression by both aggregates and individual spirochetes was performed using an anti-alginate rabbit polyclonal IgG antibody (a generous gift from Dr. G.B. Pier, Harvard University). *Borrelia burgdorferi* aggregates were grown on collagen-coated 4 well chamber slides for 7 days. The aggregates were then washed with PBS pH 7.4 and fixed in −20°C 100% methanol for 10 minutes. 1×10^7^ individual spirochete cells were collected from stock cultures and centrifuged at 8,000×g for 10 minutes at room temperature, washed once with PBS pH 7.4, and then centrifuged again at 8,000×g for 10 minutes at room temperature. The pellet was resuspended in 100 µl of 1× PBS pH 7.4, and then spread on a microscope slide (SuperFrost+, Fischer Scientific). Cells were fixed by incubating the slides in −20°C cold acetone for 10 minutes at −20°C. Slides were then washed twice with PBS pH 7.4 at room temperature. The specimens were pre-incubated with 10% normal goat serum (Thermo Scientific) in PBS/0.5% bovine serum albumin (BSA, Sigma) for 30 minutes at room temperature to block nonspecific binding of the secondary antibody. Then the primary antibody (1∶100 dilution in dilution buffer: PBS pH 7.40+0.5% BSA) was applied and the slides were incubated overnight at 4°C in a humidified chamber. After washing, specimens were incubated for 1 hour with a 1∶200 dilution of DyLight 594 Conjugated Goat Anti-Rabbit IgG (Thermo Scientific) at room temperature. The slides were then washed thrice with PBS/0.5% BSA for 10 minutes, then incubated at 37°C for 1 hour with FITC labeled *Borrelia* specific polyclonal antibody (#73005 Thermo Scientific, diluted 1∶50 in 1% BSA/1× PBS, pH 7.4). The slides were washed thrice with PBS pH 7.4 for 5 minutes at room temperature and counterstained with 4′, 6-diamidino-2-phenylindole (DAPI) for 10 minutes. The slides were then washed again with PBS pH 7.4 for 5 minutes and mounted with PermaFluor aqueous mounting medium (Thermo Scientific). Images were acquired by fluorescent microscopy. In the negative control samples, the primary antibody was omitted from slides with *Borrelia* cells and aggregates and replaced with normal rabbit sera.

### Calcium Staining Method

To determine if calcium may be involved in the composition of the film matrix, *Borrelia burgdorferi* aggregates were grown on collagen-coated 4-well chamber slides (as described above) for 7 days. The aggregates were then washed with PBS pH 7.4 and fixed with ice-cold acetone for 5 minutes. The samples were rehydrated through graded alcohol, then stained with 2% Alizarin Red-S pH 4.2 (Sigma-Aldrich #A5533) (calcium-specific stain) for four minutes at room temperature. The slides were washed twice with double distilled water and dehydrated through graded alcohols and mounted with Permount media (Fisher Scientific).

### Statistical Analysis

Statistical analyses were performed using the two sample paired t-tests with NCSS statistical software (NCSS LLC, Kaysville, UT).

## Supporting Information

Figure S1 Representative image showing the morphology of a developing *Borrelia burgdorferi* B31 aggregates by dark field microscopy. 200× magnification.
(TIF)

Figure S2 Quantitative comparison of the aggregates made by *Borrelia burgdorferi* B31 strain on different biotic and abiotic surfaces. Dilutions of 1×10^7^ B31 *Borrelia burgdorferi* cells (Lane 1∶1×10^7^; Lane 2∶5×10^6^; Lane 3∶1×10^6^; Lane 4∶5×10^5^; Lane 5∶1×10^5^; Lane 6∶5×10^4^; Lane 7∶1×10^4^; Lane 8∶5×10^3^) plated on either uncoated (Section F: polystyrene) or various matrix-coated (Section A: Matrigel, Section B: collagen, Section C: fibronectin, Section D: hyaluronan Section E: laminin) 48-well tissue culture plates. The cultures were incubated for 7 days at 33°C with 5% CO_2,_ then the colonies were stained and aggregate mass was quantified with the crystal violet method. The data represent the means of three independent experiments in which each data point was performed in triplicate. Error bars represent standard deviations.
(TIF)

Figure S3 Representative images of *Borrelia burgdorferi* B31 strain aggregates growing on various surfaces (collagen, polystyrene plastic, glass and agarose) for 7 days as described in the Materials and Methods. The aggregates were stained with either crystal violet (A–D, purple staining) or BacLight Live/Dead viability stain (E–H; green stain = live cells, red stain = dead cells). The pictures were taken at 200× magnification.
(TIF)

Figure S4 Three-dimensional AFM image of an early aggregate development of *Borrelia burgdorferi* B31 strain on mica substrate. Image produced with NanoRule© software.
(TIF)

Figure S5 Immunohistochemical staining of collagen-embedded *Borrelia burgdorferi* B31 aggregates for *Borrelia* antigen (green staining; Panels B, and F) and for alginate (red staining; Panels C and G) expression using fluorescent microscopy (see the Materials and Methods for detailed protocol). Panels A and E show dark field and Panels D and H depict DAPI-DNA counterstain images of the same aggregates. 500X magnification.
(TIF)

Video S1 A video file showing two large developing *Borrelia burgdorferi* B31 strain aggregates which are actively growing, surrounded by individual spirochetes. The file is in mp4 format.
(MP4)

Video S2 A video file showing a vertical protrusion on the surface of the *Borrelia burgdorferi* B31 stain aggregates using AFM composite images. The file is in mp4 format.
(MP4)
