# Supplementary figures and images for: Characterization of Biofilm Formation by Borrelia burgdorferi In Vitro
Source: PLoS One. 2012 Oct 24;7(10):e48277. doi: 10.1371/journal.pone.0048277 (PMC3480481; doi:10.1371/journal.pone.0048277)

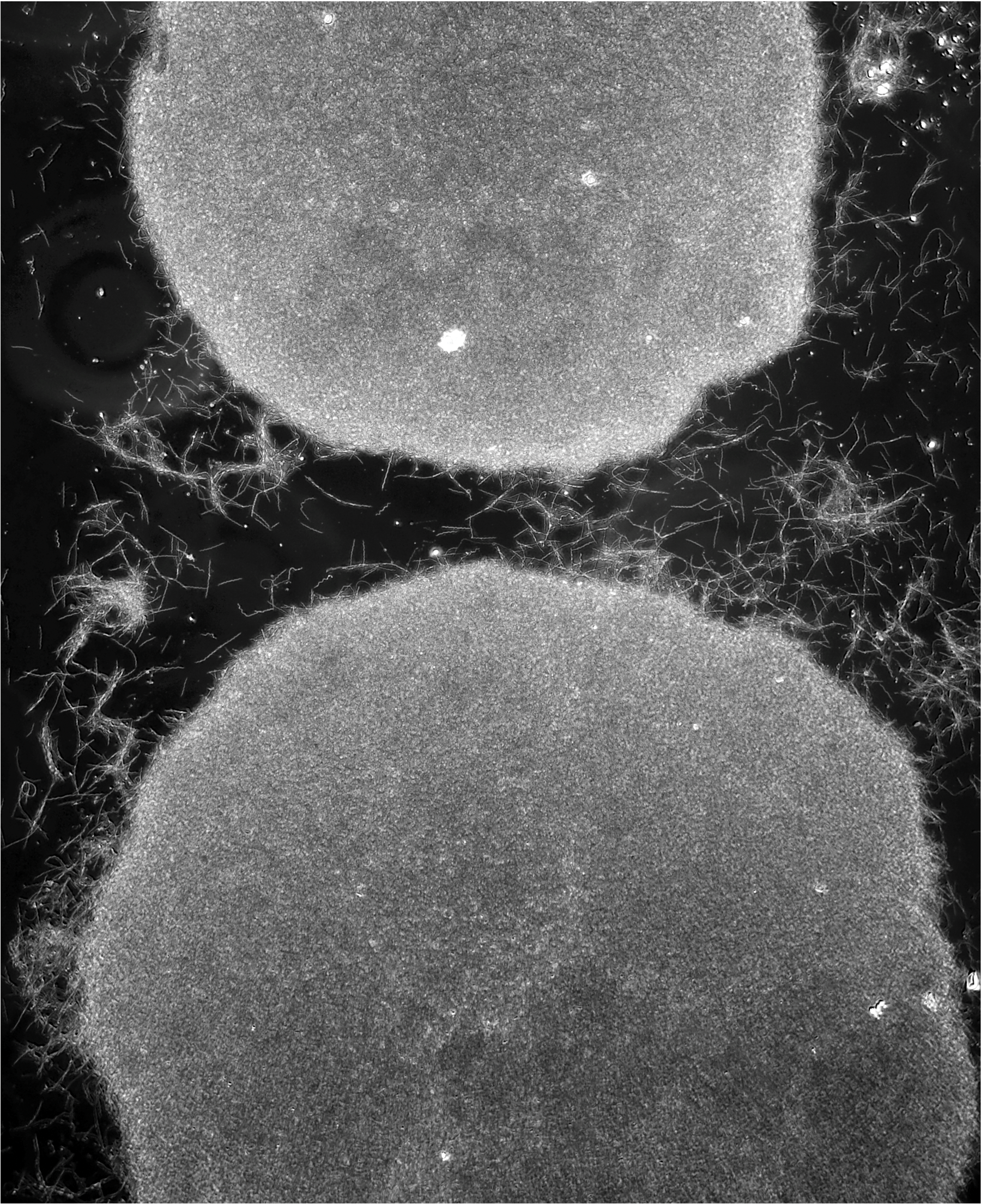

Supplement: Figure S1 — Representative image showing the morphology of a developing Borrelia burgdorferi B31 aggregates by dark field microscopy. 200× magnification. (TIF) [file pone.0048277.s001.tif]

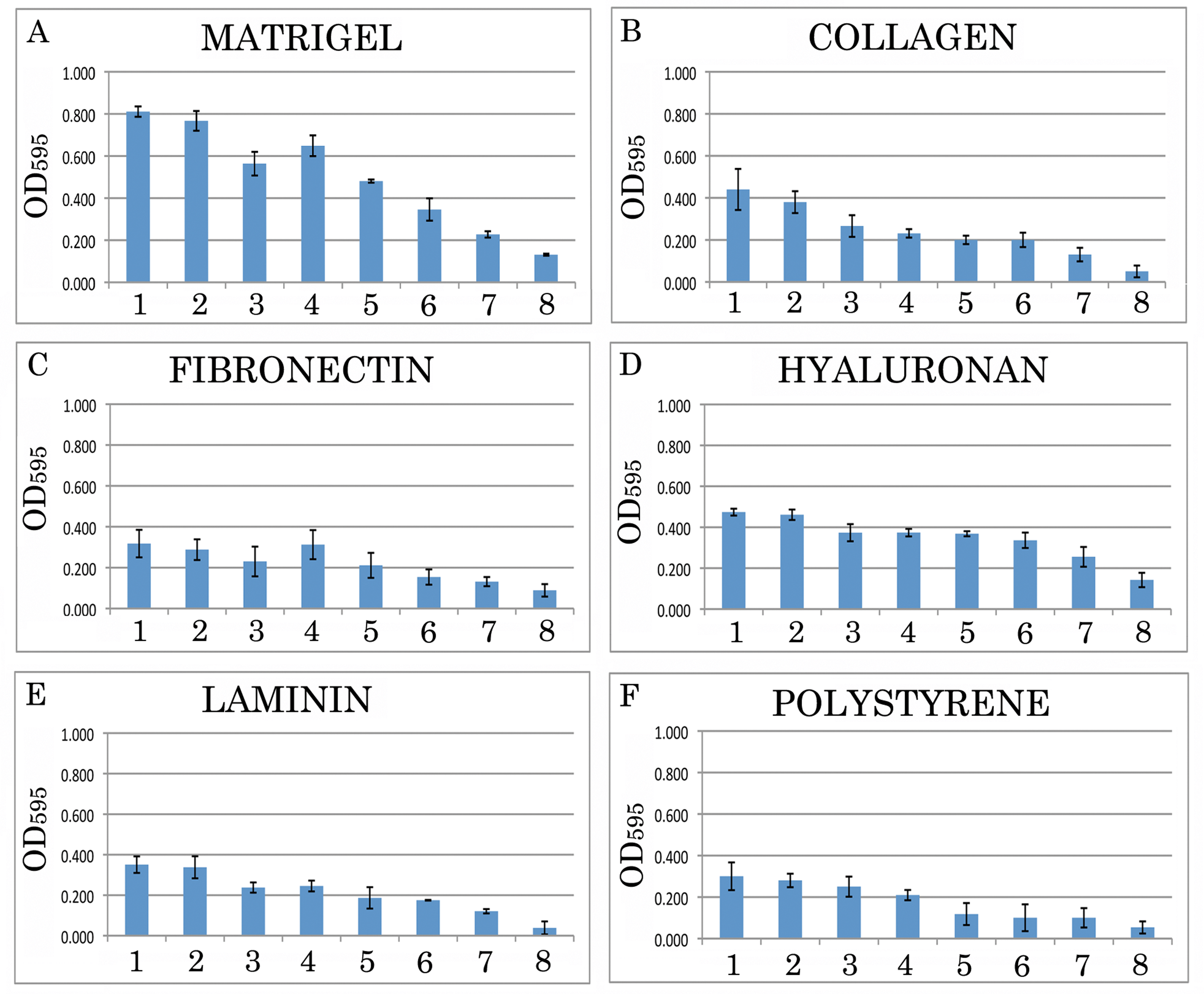

Supplement: Figure S2 — Quantitative comparison of the aggregates made by Borrelia burgdorferi B31 strain on different biotic and abiotic surfaces. Dilutions of 1×10^7^ B31 Borrelia burgdorferi cells (Lane 1∶1×10^7^; Lane 2∶5×10^6^; Lane 3∶1×10^6^; Lane 4∶5×10^5^; Lane 5∶1×10^5^; Lane 6∶5×10^4^; Lane 7∶1×10^4^; Lane 8∶5×10^3^) plated on either uncoated (Section F: polystyrene) or various matrix-coated (Section A: Matrigel, Section B: collagen, Section C: fibronectin, Section D: hyaluronan Section E: laminin) 48-well tissue culture plates. The cultures were incubated for 7 days at 33°C with 5% CO2, then the colonies were stained and aggregate mass was quantified with the crystal violet method. The data represent the means of three independent experiments in which each data point was performed in triplicate. Error bars represent standard deviations. (TIF) [file pone.0048277.s002.tif]

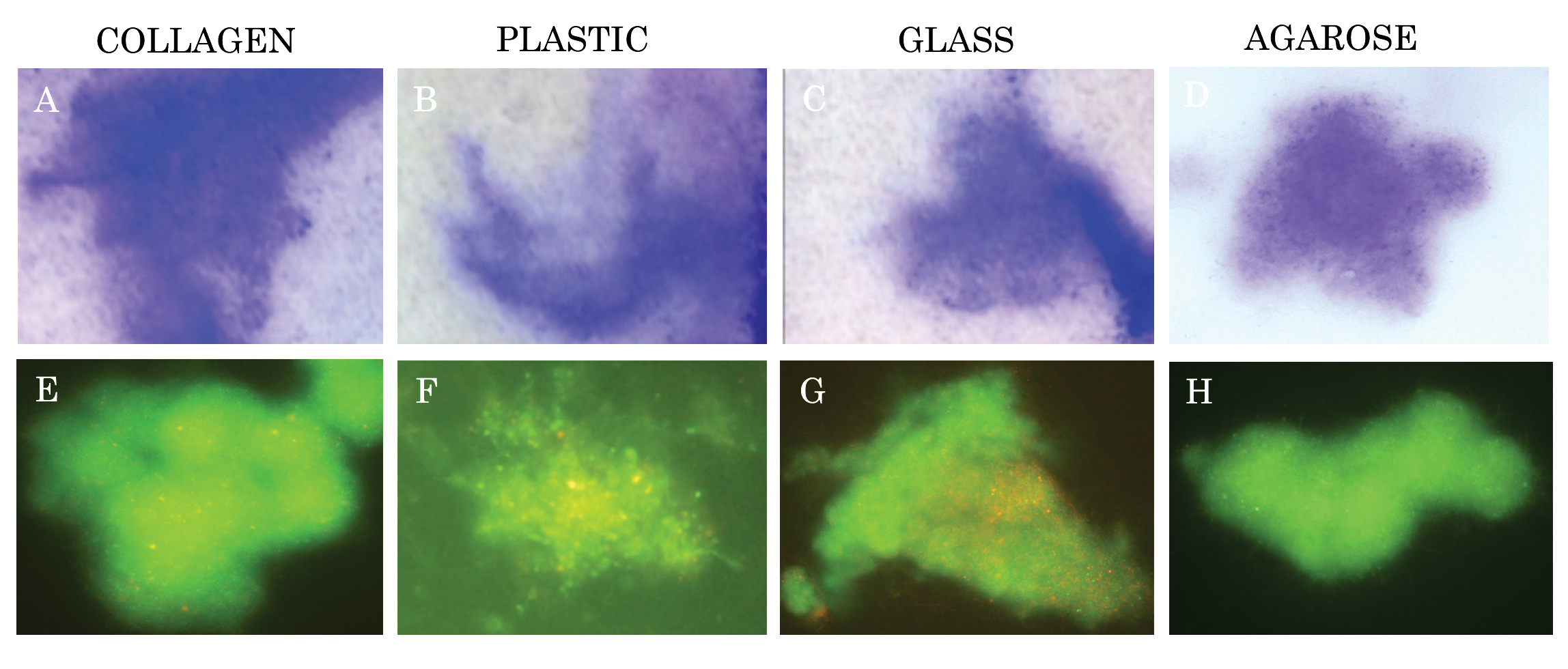

Supplement: Figure S3 — Representative images of Borrelia burgdorferi B31 strain aggregates growing on various surfaces (collagen, polystyrene plastic, glass and agarose) for 7 days as described in the Materials and Methods. The aggregates were stained with either crystal violet (A–D, purple staining) or BacLight Live/Dead viability stain (E–H; green stain = live cells, red stain = dead cells). The pictures were taken at 200× magnification. (TIF) [file pone.0048277.s003.tif]

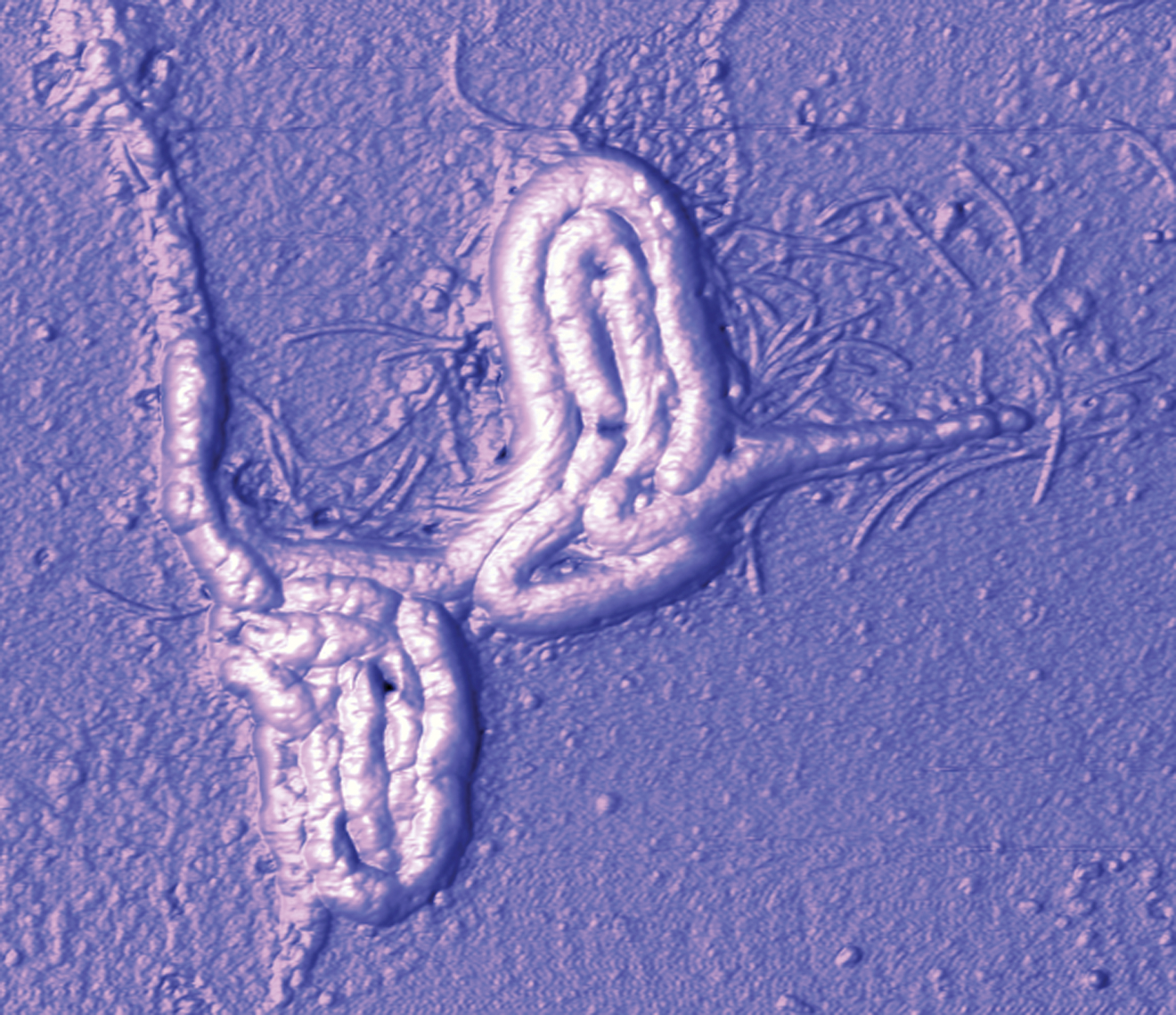

Supplement: Figure S4 — Three-dimensional AFM image of an early aggregate development of Borrelia burgdorferi B31 strain on mica substrate. Image produced with NanoRule© software. (TIF) [file pone.0048277.s004.tif]

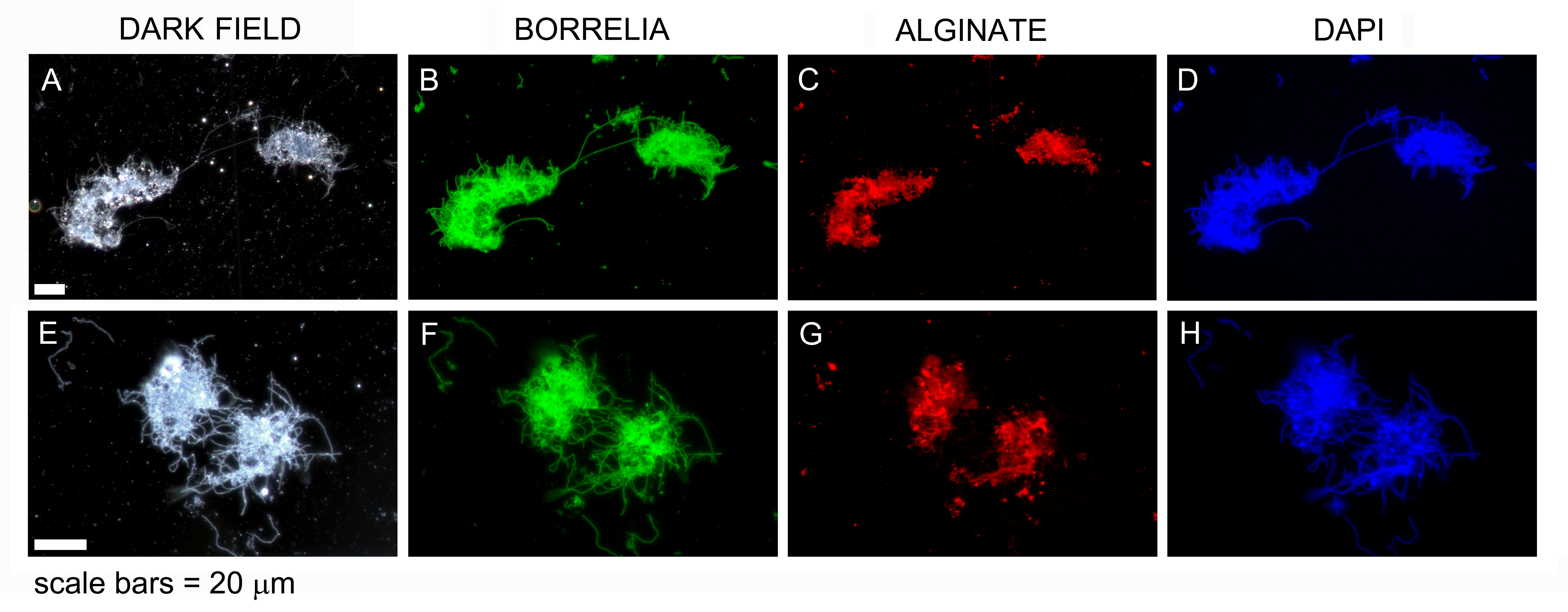

Supplement: Figure S5 — Immunohistochemical staining of collagen-embedded Borrelia burgdorferi B31 aggregates for Borrelia antigen (green staining; Panels B, and F) and for alginate (red staining; Panels C and G) expression using fluorescent microscopy (see the Materials and Methods for detailed protocol). Panels A and E show dark field and Panels D and H depict DAPI-DNA counterstain images of the same aggregates. 500X magnification. (TIF) [file pone.0048277.s005.tif]
